# Low ANXA10 expression is associated with disease aggressiveness in bladder cancer

**DOI:** 10.1038/bjc.2011.404

**Published:** 2011-10-06

**Authors:** P P Munksgaard, F Mansilla, A-S Brems Eskildsen, N Fristrup, K Birkenkamp-Demtröder, B P Ulhøi, M Borre, M Agerbæk, G G Hermann, T F Ørntoft, L Dyrskjøt

**Affiliations:** 1 Department of Molecular Medicine, Aarhus University Hospital, Skejby, Brendstrupgaardsvej 100, 8200 Aarhus N, Denmark; 2 Institute of Pathology, Aarhus University Hospital, Nbg, Nørrebrogade 44, 8000 Aarhus C, Denmark; 3 Department of Urology, Aarhus University Hospital, Skejby, Brendstrupgaardsvej 100, 8200 Aarhus N, Denmark; 4 Department of Oncology, Aarhus University Hospital, Nbg, Nørrebrogade 44, 8000 Aarhus C, Denmark; 5 Department of Urology, Frederiksberg Hospital, Copenhagen University, Ndr. Fasanvej 57, 2000 Copenhagen, Denmark

**Keywords:** bladder cancer, biomarker, progression, metastasis, ANXA10, S100A4

## Abstract

**Background:**

Markers for outcome prediction in bladder cancer are urgently needed. We have previously identified a molecular signature for predicting progression in non-muscle-invasive bladder cancer. ANXA10 was one of the markers included in the signature and we now validated the prognostic relevance of ANXA10 at the protein level.

**Methods:**

We investigated ANXA10 expression by immunohistochemistry using a tissue microarray with 249 Ta and T1 urothelial carcinomas. The expression of ANXA10 was also investigated in an additional set of 97 more advanced tumours. The functional role of ANXA10 in cell lines was investigated by siRNA-mediated ANXA10 knockdown using wound-healing assays, proliferation assays, and ingenuity pathway analysis.

**Results:**

Low expression of ANXA10 correlated with shorter progression-free survival in patients with stage Ta and T1 tumours (*P*<0.00001). Furthermore, patients with more advanced tumours and low ANXA10 expression had an unfavourable prognosis (*P*<0.00001). We found that ANXA10 siRNA transfected cells grew significantly faster compared with control siRNA transfected cells. Furthermore, a wound-healing assay showed that ANXA10 siRNA transfected cells spread along wound edges faster than control transfected cells.

**Conclusion:**

We conclude that ANXA10 may be a clinical relevant marker for predicting outcome in both early and advanced stages of bladder cancer.

Urothelial carcinoma of the urinary bladder is a common malignant disease. Patients diagnosed with bladder cancer may histologically be divided into two distinct groups with different prognosis; patients with non-muscle-invasive stage Ta and T1 tumours, often treated with a local, organ-sparing approach, and patients with muscle-invasive stages T2–T4 cancers, most often requiring immediate and more radical treatment. About 75% of patients present with non-muscle-invasive tumours initially. More than 60% of these patients experience bladder tumour recurrences and around 20% of the patients develop disease progression to a muscle-invasive bladder cancer (Millan-Rodriguez *et al*, 2000; Sylvester *et al*, 2006). Clinical risk factors associated with a high risk of disease progression to a muscle-invasive stage include deep invasion of the lamina propria, high-grade tumour, large tumour size, concurrent carcinoma *in situ* (CIS), tumour multiplicity, and recurrence of high-risk non-muscle-invasive tumours (Hermann *et al*, 1998). The remaining 25% of patients are initially diagnosed with muscle-invasive bladder cancer (stages T2–T4) and half of these patients die within 5 years (Kaufman *et al*, 2009). The standard treatment for localised stages T2–T4a tumours is radical cystectomy with lymphadenectomy. Patients with immobile tumours (T4b+) receive chemotherapy or radiotherapy – sometimes followed by salvage cystectomy (Kaufman *et al*, 2009). The use of neoadjuvant or adjuvant chemotherapy is controversial because of conflicting results (Clark, 2009); however, several studies (Grossman *et al*, 2003; Calabro and Sternberg, 2009) and recent meta-analyses (Vale, 2003; Collaboration, 2005) point in favour of both treatment regimes. Presently, no molecular biomarkers are accepted in clinical routine for selection of correct treatment regimens for patients with either non-muscle-invasive or muscle-invasive bladder cancer. In a recent study, we validated a gene expression signature in stage Ta/T1 urothelial carcinomas to be a significant predictor of progression to muscle-invasive cancer (Dyrskjot *et al*, 2007). Annexin A10 (ANXA10) was one of the markers included in the signature for predicting progression and for predicting concomitant CIS. Annexins are a family of calcium and phospholipid-binding proteins, which share a similar structure characterised by the presence of four or eight repeats of a 70-amino-acid motif and a highly variable N-terminal end (Gerke and Moss, 2002). The annexin family is composed of 12 eukaryotic members participating in diverse important biological and physiological processes including anti-coagulation, endocytosis, exocytosis, immune suppression, differentiation, and tissue growth (Gerke and Moss, 2002; Hayes and Moss, 2004; Gerke *et al*, 2005; Hayes *et al*, 2007). Many studies have shown the annexins to be among the genes consistently differentially expressed in neoplasia. Increasing evidence also indicates that cancer-specific changes in annexin expression or in their subcellular localisation contributes to development and progression of cancer by affecting cell signalling pathways, cell motility, tumour invasion and metastasis, angiogenesis, apoptosis, and drug resistance (Hayes *et al*, 2007; Mussunoor and Murray, 2008). ANXA10 expression is rare and has only been reported in liver (Liu *et al*, 2002) and in M-cells in the gastrointestinal tract (Nakato *et al*, 2009). Furthermore, ANXA10 has been reported to be correlated with poor prognosis in both hepatocellular carcinoma (Liu *et al*, 2002) and gastric carcinoma (Kim *et al*, 2009). Decreased ANXA10 has been correlated with increased invasion in a colorectal cancer cell line (Patsos *et al*, 2010) and with increased proliferation and migration in a gastric cancer cell line (Kim *et al*, 2009). Additionally, up-regulation of S100A4, which is considered a mediator of metastasis (Garrett *et al*, 2006), has been reported to down-regulate ANXA10 in a lung cancer cell line (Matsubara *et al*, 2005).

Here, we investigated the prognostic value of ANXA10 at the protein level in both non-muscle-invasive and muscle-invasive bladder cancer. Furthermore, we investigated the functional role of ANXA10 in bladder cancer cell lines by siRNA-mediated ANXA10 knockdown using wound healing, proliferation assays, and ingenuity pathway analysis (IPA).

## Materials and methods

### Patient cohorts and tumour specimens

Informed written consent was obtained from all patients, and research protocols were approved by institutional review boards or local ethical committees. Tumours from three different patient cohorts were used: Tumours from patient cohort 1: Fresh frozen non-muscle-invasive tumours used for microarray expression analysis and RT–qPCR validation. Data on these samples are published previously (Dyrskjot *et al*, 2007). Tumours from patient cohort 2: A tissue microarray containing 249 primary non-muscle-invasive tumours from patients undergoing TURB between 1979 and 2007. Only patients with a minimum of 4 years of follow-up without progression were included in the non-progressing group, in order to include patients that with a high likelihood would not have disease progression. Routine re-TURB was performed in T1 tumours where no muscle was present in the specimen. Patient follow-up time was from the first diagnostic resection to the most recent cystoscopy. Progression was defined as progression to muscle-invasive disease and was verified by pathological examination in all patients. Patients who died before progression were censured as uneventful at the time of death. Patients undergoing cystectomy before progression to muscle-invasive disease were not included on the TMA. An experienced uropathologist (BPU) reevaluated haematoxylin–eosin (HE)-stained sections according to stage and grade (WHO 2003 classification). The tissue microarray construction was done essential as described by Kononen *et al* (1998). Patients were followed by control cystoscopies in a routine schedule in accord to the Danish national guidelines, and follow-up was censored at the time of the most recent cystoscopy. Progression to muscle-invasive bladder cancer was histologically verified. Patients who underwent cystectomy before histological evidence of progression were excluded. Clinical and histopathological information is listed in Supplementary Table 1. Tumours from patient cohort 3: Localised invasive stages T1–T4a tumours from patients who received radiotherapy and cystectomy (Agerbaek *et al*, 2006). All patients received preoperative radiotherapy (40–46 Gy) followed by radical cystectomy from 1980 to 1992. None of the patients examined revealed metastatic spread to the removed lymph nodes. The tumours were graded according to the Bergkvist grading system. Detailed information about patient cohort 3 and follow-up is described in Agerbaek *et al* (2006). An overview of the three different patient cohorts is presented in Supplementary Table 2.

### RNA isolation and cDNA synthesis

Tumour tissue was frozen at −80 °C immediately after surgery and total RNA was isolated using a standard Trizol RNA extraction method (Invitrogen, Carlsbad, CA, USA). RNA quality was controlled using an Agilent Bioanalyzer (Agilent Technologies, Inc., Santa Clara, CA, USA) (criteria: RIN score>7). Total RNA was isolated from cells in culture using RNeasy mini kit (#74106; Qiagen, Valencia, CA, USA) and quality controlled using an Ultrospec 330 Pro (GE Healthcare Biosciences, Pittsburgh, PA, USA) for RT–qPCR analysis. cDNA synthesis was carried out using the SuperScript II System (Life Technologies, Carlsbad, CA, USA) (Andersen *et al*, 2004).

### Real-time RT–qPCR

RT–qPCR analysis was performed in triplicates using TaqMan probe assay (Life Technologies) ID Hs00200464_m1 (ANXA10) as recommended by the manufacturer (Applied Biosystems, Carlsbad, CA, USA) using a 7500 Fast Real-Time PCR system (Applied Biosystems). Expression levels were normalised against Ubiquitin B (UBC) expression (Andersen *et al*, 2004).

### Western blotting

All tumour specimens for western blotting were evaluated for the presence of tumour cells by HE staining. Western blotting was performed as described previously (Mansilla *et al*, 2007). We used goat polyclonal anti-ANXA10 (clone ab2343, Abcam, Cambridge, UK; 1 : 200) and polyclonal rabbit anti-goat HRP conjugated (P 0449, DakoCytomation, Glostrup, Denmark; 1 : 2000). Cell extracts from transfected COS7 and T24 cells were obtained and processed as previously described (Nordentoft *et al*, 2011). For cell extracts we used mouse anti-V5 antibody (Abcam Ltd; 1 : 2000) and goat anti-mouse HRP-conjugated antibody (Dako; 1 : 3000) and for T24 same conditions as above.

### Immunohistochemistry

Immunohistochemistry was performed on formalin-fixed paraffin-embedded 4-*μ*m sections of tumour tissue specimens transferred to Menzel Superfrost-Plus (Gerhard Menzel GmbH, Braunschweig, Germany) slides essentially as described previously (Mansilla *et al*, 2007). We used goat anti-ANXA10 antibody (ab2343, Abcam; 1 : 200–800) diluted in TBS buffer with BSA. p53 immunostaining of tumours from patient cohort 2 was performed using anti-p53 (M7001, Dako) applied in 1 : 300 in TEG-buffer.

Immunoreactivity was scored by two investigators independently. Slides were reevaluated to obtain consensus when different scores were obtained. The non-muscle-invasive tumours from patient cohort 2 were scored as follows: ANXA10 and p53; percentage of nuclear staining was defined as low (0–33%), medium (34–66%), or high (67–100%). The 97 invasive tumours from patient cohort 3 were scored slightly differently because whole-tissue sections were used for scoring. ANXA10; percentage of *ANXA10-positive regions* were defined by percentages of the cancer regions stained (either in the nucleus, in the cytoplasm, or both) as low (<10%) or high (⩾10%). p53, pRB, and S100A4 staining results were published previously on this patient cohort in Agerbaek *et al* (2003, 2006). An overview of the different analyses performed in the patient cohorts is listed in Supplementary Table 2.

### Peptide competition assay

Peptide competition assay was performed using a synthetic peptide corresponding to the N-terminal amino acids 2–14 of human ANXA10 (Abcam). The peptide was preincubated with anti-ANXA10 before immunostaining following the manufacturer's recommendation.

### Cloning and plasmid construction

Wild-type ANXA10 cDNA was PCR amplified from a commercially available plasmid containing the human ANXA10 cDNA (Origene, Rockville, MD, USA, clone TC122774) and cloned into the pcDNA 3.1 V5-His plasmid (Invitrogen) using primers sense 5′-ATCACCATGTTTTGTGGAGACTATGTG and antisense 5′-GTAGTCCTCAGCATCACCAGCA. The DNA sequence was verified by sequencing.

### Cell culture and transfections

COS7 cells were cultured in RPMI 1640 medium supplemented with 10% fetal calf serum (FCS) and 1% penicillin–streptomycin at 37 °C and 5% CO_2_ and transfected with plasmid DNA using Lipofectamine (Invitrogen) following the manufacturer's instructions. Human urinary bladder transitional cell carcinoma cell lines (T24, SW780) were cultured in DMEM medium supplemented with 10% FCS and 1% penicillin–streptomycin at 37 °C and 5% CO_2_. An siRNA pool (Dharmacon (Chicago, IL, USA) # L-012363-00) targeting ANXA10 and a control siRNA (Dharmacon # D-001206-14-20) were reverse-transfected using Lipofectamine 2000 (Invitrogen) according to the manufacturer's instructions.

### xCELLigence real-time monitoring of cell proliferation

The xCELLigence system was used according to the instructions of the supplier (Roche Applied Science, Indianapolis, IN, USA) to monitor the growth pattern of the human bladder cancer cell line SW780. In all, 6000 cells per well were reverse-transfected with siRNA (50 nM) as described above and monitored every 15 min for a period of up to 96 h by the RTCA-integrated software (Roche Applied Science) as described (Urcan *et al*, 2010). Experiments were performed twice in duplicates.

### Wound-healing assay

Cell mobility was assessed using a wound-healing assay. SW780 cells transfected with anti-ANXA10 siRNA (10 nM) were grown to confluence in 10-cm^2^ dishes (96 h post-transfection) and wounded using a sterile tip. Cells were photographed under a phase-contrast microscope after wounding and again after 30 h and the cell-free area was measured.

### Ingenuity pathway analysis

Affymetrix human exon ST 1.0 arrays were used for measuring gene expression 48 h after anti-ANXA10 siRNA transfection of SW780 and T24 cells as described previously (Thorsen *et al*, 2008). itPLIER normalisation and generation of gene expression measures was performed in GeneSpring GX 10.0 (Agilent Technologies, Inc.). Gene networks affected by anti-ANXA10 siRNA transfection were analysed by IPA software (Ingenuity Systems, Inc., Redwood City, CA, USA).

### Statistical analysis

Kaplan–Meier estimates, univariate and multivariate Cox regression analyses, *χ*^2^-tests, and logistic regression analyses were performed using the STATA 10.0 statistical analysis software (StataCorp LP, College Station, TX, USA).

## Results

**Figure 1:**
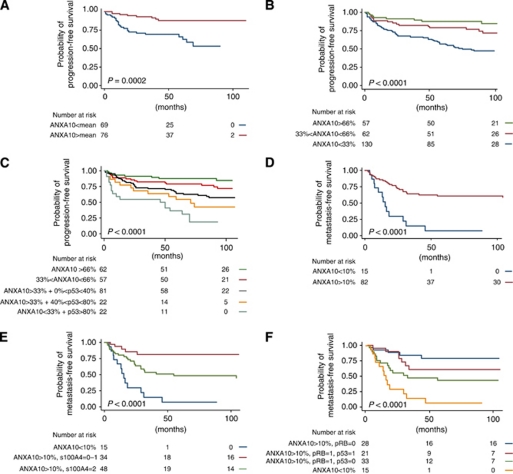
Survival as function of ANXA10 expression. (**A**) Kaplan–Meier survival plot with progression-free survival as function of *ANXA10* expression measured by microarray analysis (patient cohort 1). The patients were divided into two groups based on *ANXA10* mean expression. (**B**) Kaplan–Meier plot of progression-free survival as a function of the percentage of nuclear ANXA10 expression (*n*=249; patient cohort 2). (**C**) Kaplan–Meier plot of progression-free survival as a function of ANXA10 expression in combination with p53 nuclear immunostaining (patient cohort 2). (**D**) Kaplan–Meier plot of metastasis-free survival as a function of *ANXA10-positive regions* (patient cohort 3). (**E**) Kaplan–Meier plot of metastatic-free survival as a function of both *ANXA10-positive regions* and S100A4 focal staining (patient cohort 3). (**F**) Kaplan–Meier plot of metastasis-free survival as a function of *ANXA10-positive regions*, nuclear staining of p53, and nuclear staining of pRB (patient cohort 3).

The *ANXA10* gene expression levels in tumours from 150 patients were reported previously (Dyrskjot *et al*, 2007). A gene expression signature including ANXA10 was validated as being able to predict the presence of concomitant CIS, and progression to muscle-invasive bladder cancer. In the present study, we focused on the *ANXA10* gene expression and in patient cohort 1, we found a 3.2-fold higher *ANXA10* expression in tumours without concomitant CIS compared to tumours with concomitant CIS (*P*=0.000002, *t*-test). Furthermore, low expression of *ANXA10* correlated with shorter progression-free survival (Figure 1A). Microarray measurement of *ANXA10* mRNA expression was successfully validated by RT–qPCR (Pearson correlation: 0.9; data not shown).

**Figure 2:**
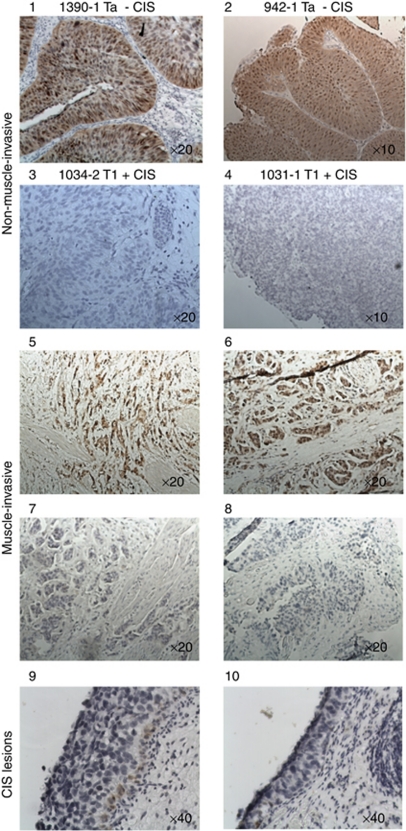
ANXA10 expression in tumour tissue and CIS lesions. (1–4) Non-muscle-invasive bladder tumours with either high (1, 2) or low (3, 4) expression of ANXA10. (5–8) Muscle-invasive bladder tumours with either high (4–6) or low (7, 8) expression of ANXA10. (9, 10) ANXA10 expression in CIS lesions.

A high expression of ANXA10 in tumours without concomitant CIS compared to tumours with concomitant CIS was also shown at the protein level by western blotting (Supplementary Figure 1) and immunostaining (Figure 2). Immunostaining revealed strong but heterogeneous nuclear staining and medium cytoplasmic staining of ANXA10 in tumours without concomitant CIS and weak or no staining in tumours with concomitant CIS and in CIS lesions. Antibody specificity was validated by western blotting and by peptide competition assays (Supplementary Figures 2 and 3).

### Expression of ANXA10 in non-muscle-invasive bladder cancer

For validation of the prognostic value of ANXA10 at the protein level, we immunostained a bladder cancer tissue microarray. The patients had a median age of 68 years (range: 32–86 years) and the median follow-up time was 76 months (range: 1–232 months). Further clinical and histopathological information is listed in Supplementary Table 1. Acceptable inter-observer agreement was obtained between the independent scoring of the staining (anti-ANXA10, *κ*=0.82; anti-p53, *κ*=0.73). ANXA10 immunostaining revealed that 130 of 249 cases (52%) showed low, 62 cases (25%) medium, and 57 cases (23%) high ANXA10 nuclear expression. Low ANXA10 expression was significantly associated with a shorter progression-free survival (*P*<0.00001, log rank test; Figure 1B). Univariate Cox proportional hazards analysis of progression-free survival showed that ANXA10 and established prognostic factors including tumour stage, histological grade, age, and tumour growth pattern were significant risk factors for progression (Table 1). Multivariate analysis showed that low ANXA10 expression was an independent predictor of progression-free survival (HR: 0.35; *P*=0.003). In addition, we found a highly significant association between tumours with concomitant CIS and low ANXA10 expression (*P*<0.0001, *χ*^2^-test).

Down-regulation of ANXA10 and p53-positive immunostaining has been described to act synergistically towards high-grade and high-stage cancer and poor prognosis in hepatocellular cancer (Liu *et al*, 2002). We therefore investigated the prognostic value of p53 alone and together with ANXA10. p53 immunostaining was significantly associated with shorter progression-free survival (*P*<0.0001, log rang test; Supplementary Figure 4A). Furthermore, an inverse correlation between ANXA10 and p53 expression was observed (*P*<0.02, *χ*^2^-test; Supplementary Figure 4B). Combining ANXA10 and p53 expression resulted in a highly significant prediction of progression (*P*<0.00001, log rank test; Figure 1C).

### ANXA10 expression in muscle-invasive bladder cancer

We also investigated the prognostic value of ANXA10 in more advanced tumours. The clinical and histopathological information for all patients is listed in Table 2. The patients had a median age of 62 years (range: 46–73 years) and the median follow-up time was 24 months (range: 1–242 months). In this patient cohort, 15 of 97 patients (15%) had tumours with low ANXA10 expression, whereas 82 patients (85%) showed high ANXA10 expression. We observed that in the ANXA10-positive tumours, the cancer cells generally revealed a strong but heterogeneous nuclear staining and medium cytoplasmic staining, similar to the non-muscle-invasive tumours (Figure 2). Kaplan–Meier survival curves showed that patients with low ANXA10 expression had an unfavourable prognosis (*P*<0.00001, log rank test; Figure 1D). Metastatic disease was the only clinical variable that was significantly associated with ANXA10 expression in muscle-invasive tumours (Table 2). Furthermore, low ANXA10 expression was significantly associated with metastatic-free survival in univariate Cox regression analysis (HR: 0.24 (95% confidence interval: 0.12–0.48); *P*<0.001). All other clinical and histopathological parameters showed no correlation to survival (data not shown).

ANXA10 has been described to be down-regulated in response to up-regulation of S100A4 in a lung cancer cell line (Matsubara *et al*, 2005). Furthermore, strong focal staining of S100A4 has been shown to act as prognostic marker for metastatic disease in bladder cancer (Agerbaek *et al*, 2006). We therefore investigated the inverse correlation between S100A4 and ANXA10 expression. Interestingly, 14 out of 15 patients with low ANXA10 expression were S100A4 positive, compared to 66 out of 82 with high ANXA10 expression. Furthermore, combining ANXA10 and S100A4 expression was a highly significant predictor of metastatic disease (*P*<0.00001, log rank test; Figure 1E).

When analysing p53 expression in the muscle-invasive tumours, we found that 10 out of the 15 patients with low ANXA10 expression were p53 positive. We found an inverse correlation between p53 and ANXA10 expression in non-muscle-invasive tumours; however, this was not the case in muscle-invasive cancers (*P*<0.3, *χ*^2^-test). In this patient cohort, complete response to radiotherapy was associated with positive p53 immunostaining (*P*<0.04, *χ*^2^-test; Supplementary Figure 4C) and as a consequence, positive p53 immunostaining was a significant predictor of cancer-free survival (*P*=0.01, log rank test; Supplementary Figure 5A). Retinoblastoma (RB) protein expression has been reported to be an independent predictor of both response to radiotherapy and survival in this patient cohort (Agerbaek *et al*, 2003). Here, we only had access to 97 out of the original 108 patients, and here we found that RB expression was only a borderline significant predictor of cancer-free survival (*P*<0.07, log rank test; Supplementary Figure 5B). However, combining ANXA10, p53, and RB expression resulted in a highly significant prediction of cancer-free and metastatic-free survival (*P*<0.00001, log rank test; Figure 1F).

### ANXA10 down-regulation affects proliferation and migration in a bladder cancer cell line

**Figure 3:**
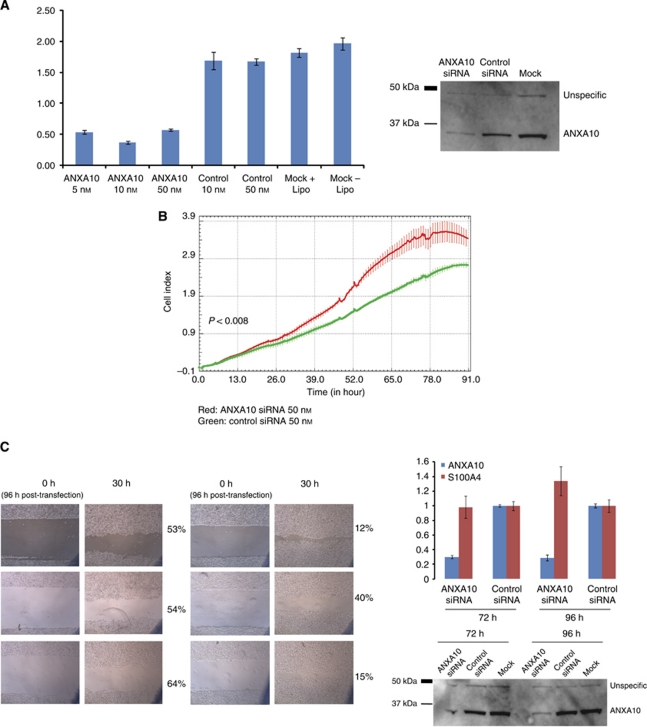
Phenotypic effects of siRNA-mediated knockdown of ANXA10. (**A**) ANXA10 knockdown in the bladder cancer cell line SW780 was validated by RT–pPCR (left) and western blotting analysis (right) at 48 h post-transfection. Cells were transfected with 5, 10, 50 nM of ANXA10 (ANXA10), 10 nM Control siRNA (Control), no siRNA with transfection reagent (Mock+Lipo), or no siRNA or transfection reagent (Mock). A concentration of 10 nM siRNA was used for western blotting (right). Both experiments were performed in duplicate. (**B**) The SW780 bladder cancer cell line was transfected with 50 nM siRNA against either ANXA10 (purple) and 50 nM control siRNA (red). The cell index (CI) describes the degree of cell confluence. (**C**) Wound-healing experiments were performed by wounding confluent SW780 cells 96 h post-transfection with either anti-ANXA10 siRNA or control siRNA (left). ANXA10 and S100A4 expression were measured by RT–qPCR and normalised to Ubiquitin B expression at 72 and 96 h post-transfection (right). Control samples were normalised to 1. ANXA10 knockdown was validated by western blotting analysis at 72 and 96 h post-transfection.

We studied the phenotypic effects in a SW780 bladder cancer cell line, endogenously expressing ANXA10. We examined whether ANXA10 affected cell proliferation and migration by transfection with a pool of siRNA (Dharmacon) targeting ANXA10 and a control siRNA. We achieved an ∼75% siRNA-mediated ANXA10 knockdown, which was validated by RT–qPCR and by western blot analysis 48 h post-transfection (Figure 3A). We found that cell growth in ANXA10 siRNA transfected cells was significantly faster compared with control siRNA transfected cells (Figure 3B). Next, the effect of ANXA10 on cell motility was assessed using a wound-healing assay (Figure 3C). ANXA10 siRNA transfected cells spread along wound edges faster than control transfected cells, indicating that down-regulation of ANXA10 may increase cell migration. Down-regulation of ANXA10 has been reported in response to up-regulating of S100A4 in a lung cancer cell line (Matsubara *et al*, 2005). We showed that down-regulation of ANXA10 caused up-regulation of S100A4 in the SW780 bladder cancer cell line (Figure 3C). The phenotypic effect of ANXA10 knockdown was investigated further by profiling gene expression 48 h after transfection of SW780 and T24 cells with 10 nmol l^–1^ anti-ANXA10 siRNA or scrambled sequence. Gene expression microarray data were analysed using IPA software and gene networks were generated from gene expression changes 48 h following transfection. The results obtained from IPA identify cellular networks of identified molecules and not molecular pathways. However, the top five gene networks were associated with the functional categories: cellular growth and proliferation (SW780 and T24), cell signalling (SW780 and T24), cell-to-cell signalling and interaction (SW780), cellular movement (T24), and cell cycle (T24).

## Discussion

We showed that low ANXA10 mRNA expression was associated with the presence of concomitant CIS and a shorter progression-free survival. Furthermore, we found that ANXA10 immunostaining independently was able to stratify patients at risk for disease progression in non-muscle-invasive bladder tumours and at risk of developing metastasis in muscle-invasive cancers. Finally, we found that down-regulation of ANXA10 in a bladder cancer cell line induced increased proliferation and migration.

Standard treatment of localised muscle-invasive bladder cancer (stages T2–T4a) is cystectomy with pelvic lymphadenectomy. Identification of patients with high risk of micro-metastases by biomarkers as, for example, ANXA10 may facilitate usage of neoadjuvant chemotherapy both as early treatment of metastasis and as treatment of metastatic disease while the burden of disease is low. The patient cohort studied here has been subjected to presurgical radiotherapy, which is not a standard treatment today. However, since no correlation between ANXA10 expression and response to radiation therapy was found (data not shown) our results indicate that low ANXA10 expression is a marker for metastasis in muscle-invasive bladder cancer.

Interestingly, the prognostic value of ANXA10 was independent of disease stage in both non-muscle-invasive and muscle-invasive bladder cancer. Consequently, we observed that the frequency of tumours with low expression of ANXA10 in the two patient cohorts differed significantly with relatively fewer tumours with low expression in the muscle-invasive patient cohort than expected if the marker was stage dependent. Some of the differences in ANXA10 expression frequency may also be caused by the difference in scoring between the two patient cohorts.

Interestingly, ANXA10 and p53 expression has been reported to be inversely correlated to each other and to clinical outcome in patients in hepatocellular cancer (Liu *et al*, 2002). The p53 protein has a very short half-life and mutation of p53 stabilises the protein, which makes it detectable by immunostaining. In our study, positive p53 immunostaining was associated with shorter progression-free survival in the non-muscle-invasive tumours. We further observed an inverse correlation between low ANXA10 expression and high p53 expression. The combination of ANXA10 and p53 expression resulted in a strong prediction of progression to muscle-invasive cancer. This is in agreement with a previous study in hepatocellular carcinoma where ANXA10 and p53 expression revealed significant inverse correlation and down-regulation of ANXA10 and p53 mutation acted synergistically towards poorer prognosis (Liu *et al*, 2002).

In muscle-invasive bladder cancer, positive p53 immunostaining was associated with longer cancer-free survival. This opposite prognostic value of p53 in the muscle-invasive tumours, compared with the non-muscle-invasive tumours, may be explained by the fact that positive p53 immunostaining was associated with a high rate of complete response to radiotherapy. This means that mutations in p53 are beneficial for response to radiotherapy as DNA damage would not be repaired and thereby overall patient survival would be improved. Furthermore, loss of pRB expression is normally regarded as a prognostic marker for poor survival in bladder cancer (Cordon-Cardo *et al*, 1992, 1997; Logothetis *et al*, 1992). However, immunostaining with pRB of the same patient cohort as in our study revealed that negative pRB was significantly associated with high response to radiotherapy and thereby cancer-free survival (Agerbaek *et al*, 2003).

Our data are based on retrospective analyses, which may have some limitations as potential bias in patient selection and changes in treatment strategies over time. As an example, all patients in cohort 3 received preoperative radiotherapy, which is not a standard treatment today. Therefore, the predictive value of ANXA10 needs to be validated in prospective studies before clinical implementation both to verify the predictive value of ANXA10 and to determine the optimal cutoff values.

Several annexins and S100 proteins are known to interact and form complexes that exhibit biological activities (Moss and Morgan, 2004; Gerke *et al*, 2005; Miwa *et al*, 2008). Our study indicates an association between expression levels of ANXA10 and S100A4 in bladder cancer. First, ANXA10 has been reported to be down-regulated in response to up-regulation of S100A4 in a lung cancer cell line (Matsubara *et al*, 2005). We showed that down-regulation of ANXA10 in a bladder cancer cell line (SW780) resulted in up-regulation of S100A4. Next, strong focal staining of S100A4 has been associated with the development of metastatic disease and thereby poor prognosis in muscle-invasive bladder cancer (Agerbaek *et al*, 2006). We showed that low ANXA10 expression was associated with the development of metastatic disease. Furthermore, we improved the prognostic value considerably by combining S100A4 with ANXA10 expression, which resulted in a strong and independent prediction of metastatic disease in patients with muscle-invasive cancer. Animal and cellular studies implicating S100A4 in the establishment of a metastatic phenotype are numerous (reviewed in Garrett *et al*, 2006). Up-regulation of S100A4 in an invasive but non-metastatic rat bladder cancer cell line generates metastatic variants (Levett *et al*, 2002). Furthermore, S100A4 has been shown to have prognostic value in a number of human cancers such as breast, oesophageal-squamous, non-small cell lung, primary gastric, malignant melanomas, prostate and pancreatic carcinomas (Garrett *et al*, 2006), besides bladder cancer (Davies *et al*, 2002; Agerbaek *et al*, 2006).

In our study, down-regulation of ANXA10 in a bladder cancer cell line resulted in increased proliferation and migration. This is in agreement with earlier cell line studies, revealing that decreased ANXA10 was correlated with increased invasion in a colorectal cancer cell line (Patsos *et al*, 2010) and with increased proliferation and migration in a gastric cancer cell line (Kim *et al*, 2009). The biological role of ANXA10 is not clear since ANXA10 deviate from the annexin family by having only one functional Ca^2+^-binding motif. How this affects the membrane-binding or membrane aggregation properties and thereby the function of ANXA10 is unclear. Interestingly, we observed that down-regulation of ANXA10 induced up-regulation of S100A4. These data indicate that the metastatic and invasive phenotype associated with low ANXA10 expression may be associated with the concomitant up-regulation of S100A4, which is a well-known inducer of invasion and metastasis.

We conclude that ANXA10 may be a clinical relevant marker for predicting outcome in both early and advanced stages of bladder cancer. Furthermore, ANXA10 may be directly involved in regulating cell growth and cell migration.

## Figures and Tables

**Table 1:** Univariate and multivariate Cox regression analysis of progression-free survival for patients with non-muscle-invasive tumours

		**Univariate analysis**		**Multivariate analysis**	
**Variables**	**Categorisation**	**Hazard ratio**	***P*-value**	**Hazard ratio**	***P*-value**
Age	5-year interval	1.18 (1.06–1.32)	0.003	1.09 (0.97–1.22)	0.128
Sex	Male *vs* female	0.92 (0.56–1.51)	0.755		
Tumour stage	Ta *vs* T1	2.58 (1.73–3.85)	<0.0001	0.82 (0.33–2.00)	0.661
Histological grade	PUNLMP/low *vs* high	3.02 (2.02–4.51)	<0.0001	2.92 (1.17–7.28)	0.021
Tumour size	<3 *vs* >3 cm	0.97 (0.60–1.56)	0.900		
BCG treatment	No BCG *vs* BCG	0.57 (0.32–1.01)	0.055	0.47 (0.25–0.89)	0.020
Concomitant CIS^a^	No CIS *vs* CIS	1.41 (0.94–2.12)	0.098	1.13 (0.71–1.80)	0.601
Growth pattern	Papillary *vs* solid	2.71 (1.40–5.25)	0.003	1.56 (0.79–3.09)	0.201
ANXA10 nuclear %	Low *vs* medium	0.49 (0.30–0.80)	0.005	0.59 (0.34–1.02)	0.058
	Low *vs* high	0.25 (0.13–0.50)	<0.0001	0.35 (0.17–0.70)	0.003

Abbreviation: BCG=Bacillus Calmette-Geurin; CIS=carcinoma *in situ*; PUNLMP=papillary urothelial neoplasm of low malignant potential.

a Three samples without information about CIS status were excluded.

Only variables with *P*<0.1 in univariate analysis were included in the multivariate analysis.

**Table 2:** Association between ANXA10 expression and clinical variables in advanced cancers

**Characteristics**	**Categorisation**	**Number**	**ANXA10⩽10%**	**ANXA10>10%**	***P*-value, *χ*^2^-test**
All patients		97	15 (15%)	82 (85%)	
Age	⩽Median (62 years)	55	9 (16%)	46 (84%)	
	>Median	42	6 (14%)	36 (86%)	0.779
Sex	Female	16	3 (19%)	13 (81%)	
	Male	81	12 (15%)	69 (85%)	0.691
Tumour stage	T1	4	1 (25%)	3 (75%)	
	T2	25	5 (20%)	20 (80%)	
	T3a	25	4 (16%)	21 (84%)	
	T3b	32	5 (16%)	27 (84%)	
	T4a	11	0 (0%)	11 (100%)	0.611
Histological grade	II	1	0 (0%)	100 (100%)	
	III	76	12 (16%)	64 (84%)	
	IV	20	3 (15%)	17 (85%)	0.908
Concomitant CIS	Yes	32	4 (13%)	28 (87%)	
	No	36	6 (17%)	30 (83%)	
	Unspecified	29	5 (17%)	24 (83%)	0.850
Response to radiotherapy^a^	Complete response (CR)	39	6 (15%)	33 (85%)	
	Incomplete response (IR)	57	9 (16%)	48 (84%)	0.957
Histology	Pure TCC	78	14 (18%)	64 (82%)	
	TCC with squamous differentiation	2	0 (0%)	2 (100%)	
	TCC with adenomatous differentiation	17	1 (6%)	16 (94%)	0.381
Metastatic relapse	Yes	41	13 (32%)	28 (68%)	
	No	56	2 (4%)	54 (96%)	<0.001
Relapse	Yes	48	13 (27%)	35 (73%)	
	No	49	2 (4%)	47 (96%)	0.002

Abbreviation: CIS=carcinoma *in situ*; TCC=transitional cell carcinoma; TURB=Transurethral resection of the bladder.

a CR: absence of residual malignant cells or IR: presence of residual malignancy in the cystectomy specimen. One patient was not evaluable for response to radiotherapy because of microscopically radical TURB.
