# Supplementary material for: Low ANXA10 expression is associated with disease aggressiveness in bladder cancer
Source: Br J Cancer. 2011 Oct 6;105(9):1379–87. doi: 10.1038/bjc.2011.404 (PMC3241563; doi:10.1038/bjc.2011.404)

**Supplementary Information**

**Low ANXA10 expression is associated with disease aggressiveness in bladder cancer**

Pia Pinholt Munksgaard^1^, Francisco Mansilla^1^, Anne-Sofie Brems Eskildsen^1^, Niels Fristrup^1^, Karin Birkenkamp-Demtröder^1^, Benedicte Parm Ulhøi^2^, Michael Borre^3^, Mads Agerbæk^4^, Gregers G. Hermann^5^, Torben F. Ørntoft^1^ and Lars Dyrskjøt^1^.

^1^Department of Molecular Medicine, Aarhus University Hospital, Skejby, Brendstrupgaardsvej 100, 8200 Aarhus N, Denmark; ^2^Institute of Pathology, Aarhus University Hospital, Nbg, Nørrebrogade 44, 8000 Aarhus C, Denmark; ^3^ Department of Urology, Aarhus University Hospital, Skejby, Brendstrupgaardsvej 100, 8200 Aarhus N, Denmark; ^4^Department of Oncology, Aarhus University Hospital, Nbg, Nørrebrogade 44, 8000 Aarhus C, Denmark; ^5^ Department of Urology, Frederiksberg Hospital, Copenhagen University, Ndr. Fasanvej 57, 2000 Copenhagen, Denmark

Supplementary Table 1

Clinical and histopathological characteristics of patients with primary non-muscle invasive urothelial bladder cancer and correlation to ANXA10 protein expression.

| Characteristics | Number | Number  ANXA10  0-33%* | Number  ANXA10  34-66%* | Number ANXA10  67-100%* | ***P-value***  Chi^2^ test |
| --- | --- | --- | --- | --- | --- |
| **All patients** | 249 |  |  |  |  |
| **Sex** |  |  |  |  |  |
| Women | 54 | 21 (39%) | 15 (28%) | 18 (33%) |  |
| Men | 195 | 109 (56%) | 47 (24%) | 39 (20%) | 0.053 |
| **Tumor stage** |  |  |  |  |  |
| Ta | 161 | 67 (42%) | 48 (30%) | 46 (28%) |  |
| T1 | 88 | 63 (72%) | 14 (16%) | 11 (12%) | <0.001 |
| **Histological grade** |  |  |  |  |  |
| PUNLMP + Low grade | 161 | 64 (40%) | 51 (32%) | 46 (28%) |  |
| High grade | 88 | 66 (75%) | 11 (13%) | 11 (13%) | <0.001 |
| **Tumor size** |  |  |  |  |  |
| < 3 cm | 151 | 82 (54%) | 34 (23%) | 35 (23%) |  |
| > 3 cm | 69 | 32 (46%) | 20 (29%) | 17 (25%) |  |
| Unspecified | 29 | 16 (55%) | 8 (28%) | 5 (17%) | 0.732 |
| **Growth pattern** |  |  |  |  |  |
| Papillary | 220 | 108 (49%) | 57 (26%) | 55 (25%) |  |
| Solid | 16 | 14 (86%) | 1 (6%) | 4 (31%) |  |
| Mixed | 13 | 8 (61%) | 4 (31%) | 1 (8%) | 0.031 |
| **Concomitant CIS** |  |  |  |  |  |
| Yes | 78 | 53 (67%) | 18 (23%) | 7 (9%) |  |
| No | 168 | 76 (45%) | 42 (25%) | 50 (30%) |  |
| Unspecified | 3 | 1 (33%) | 2 (67%) | 0 (0%) | 0.001 |
| **BCG treatment** |  |  |  |  |  |
| Yes | 46 | 26 (56%) | 13 (28%) | 7 (15%) |  |
| No | 203 | 104 (51%) | 49 (24%) | 50 (25%) | 0.385 |

* nuclear immunostaining.

Supplementary Table 2

| Cohort # | # of patients | All analyses performed | Analyses performed in this study | Included analyses from previous studies |
| --- | --- | --- | --- | --- |
| Patient cohort 1 | 150 | ANXA10 microarray and rt-q-PCR data | ANXA10 rt-q-PCR | ANXA10 microarray data |
| Patient cohort 2 | 249 | ANXA10 and p53 IHC | ANXA10 and p53 IHC | none |
| Patient cohort 3 | 97 | ANXA10, p53, pRB and S100A4 IHC | ANXA10 and P53* IHC | P53*, pRB and S100A4 IHC |

* p53 immunostaining and scoring of patient cohort 3 was performed by Agerbæk M et al. together with pRB and S100A4, but p53 was not published in the previous study. We therefore include the p53 data in this manuscript.

Supplementary Figure 1

Western blot of nine bladder tumors (Ta/T1) selected based on microarray expression data – 5 tumors with high (-CIS) and 4 tumors with low expression (+CIS) of ANXA10. ANXA10 is observed at 32 kDa.

* defines an unspecific band. This band correlates with the expression of beta-actin and is hence used as loading control in Fig. 3A and Fig. 3C.

Supplementary Figure 2

Overexpression of ANXA10-V5 in COS7 cell line and endogenous expression of ANXA10 in a bladder cancer cell line. Western blot analysis of COS7 cells transfected with either an ANXA10-V5 (A10-V5) construct or an empty construct (Mock) revealed an unspecific band at 48 kDa and a strong band at 37 kDa representing ANXA10-V5. The bladder cancer cell line T24 revealed both the unspecific band and a band around 32 kDa representing endogenous ANXA10. The V5-tag corresponds to 5kDa, which explains the difference between the overexpressed and the endogenous ANXA10.


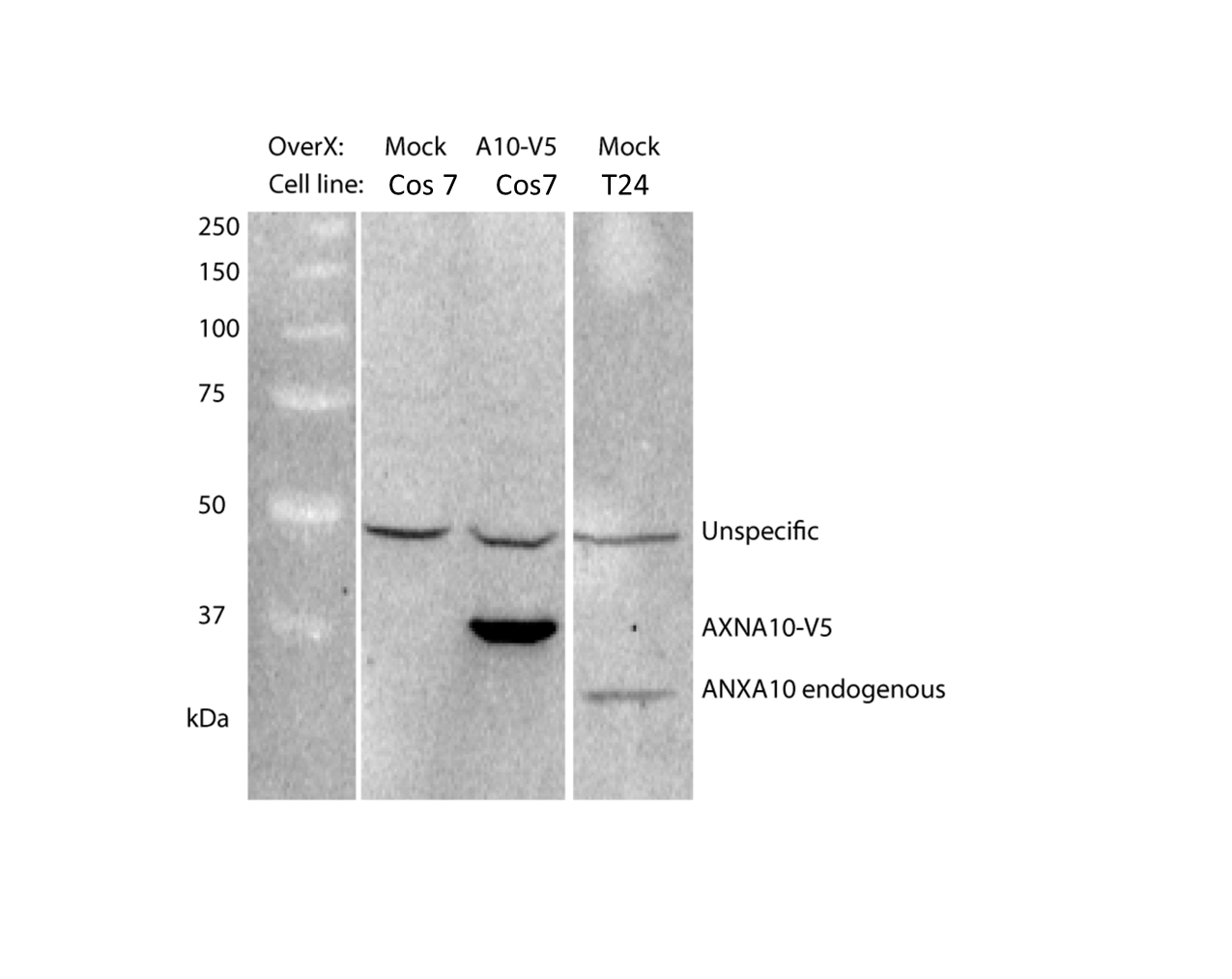


Supplementary Figure 3

Peptide competition assay using a synthetic peptide corresponding to the N terminal amino acids 2-14 of human ANXA10 (from Abcam).


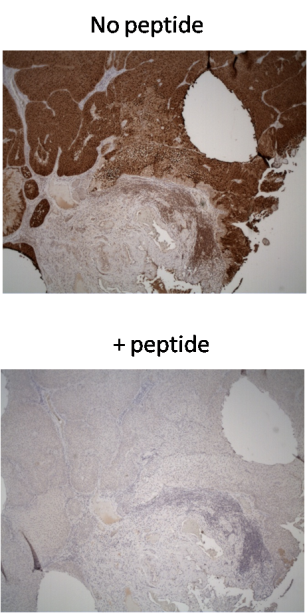


Supplementary Figure 4

p53 immunostaining in bladder cancer. A) Kaplan-Meier plot of progression-free survival as a function of the percentage of nuclear p53 expression (0-40%, 40-80%,80-100%) in a cohort of 241 Ta/T1 tumors. B) Correlation between ANXA10 and p53 expression (0=<20% nuclear staining, 1=>20% nuclear staining). C) Correlation between p53 expression and response to radiation therapy. Only 96 patients were included because one patient was not evaluable for response to radiotherapy because of microscopically radical TUR-B.

A **Non-muscle-invasive bladder cancer (Ta/T1)**

**
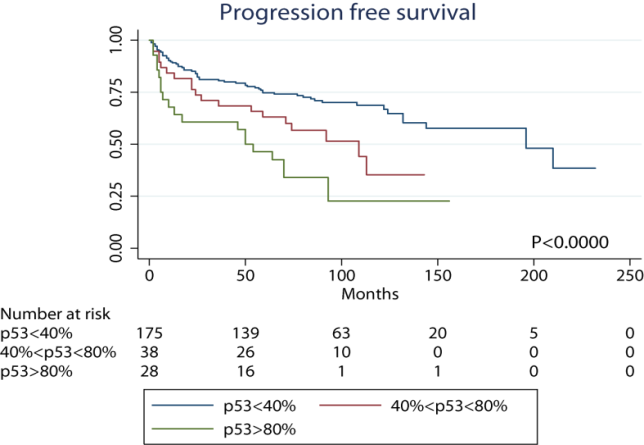
**

B **Non-muscle-invasive bladder cancer (Ta/T1)**

|  | ANXA10 nuclear percentage | | | | |
| --- | --- | --- | --- | --- | --- |
| p53 nuclear percentage | 0-33% | 33-67% | 67-100% | Total |  |
| 0-40% | 81  64.8% | 48  78.69% | 46  83.64% | 175  72.61% |  |
| 40-80% | 22  17.60% | 10  16.39% | 6  10.91% | 38  15.77% |  |
| 80-100% | 22  17.60% | 3  4.92% | 3  5.45% | 28  11.625 |  |
| Total | 125  100% | 61  100% | 55  100% | 241  100% |  |

Chi^2^ - P=0.023

C **Invasive bladder cancer (T1-T4a)**

|  | Complete response to radiation therapy | | |
| --- | --- | --- | --- |
| P53 | no | yes | Total |
| 0 | 31  54% | 13  33% | 44  46% |
| 1 | 26  46% | 26  67% | 52  54% |
| Total | 57  100% | 39  100% | 96  100% |

Chi^2^ - P=0.042

Supplementary Figure 5

p53 and pRB are predictive markers for disease relapse in muscle-invasive cancer – alone and together with ANXA10. A) Kaplan-Meier plot of cancer-free survival as a function of the percentage of “ANXA10 positive regions”, p53 nuclear staining (0: no staining and 1: nuclear staining) or combined. B) Kaplan-Meier plot of cancer-free survival as a function of the percentage of “ANXA10 positive regions”, pRB staining (0: no staining and 1: nuclear staining) or combined.

The number of patients in each group is shown on the graphs

A


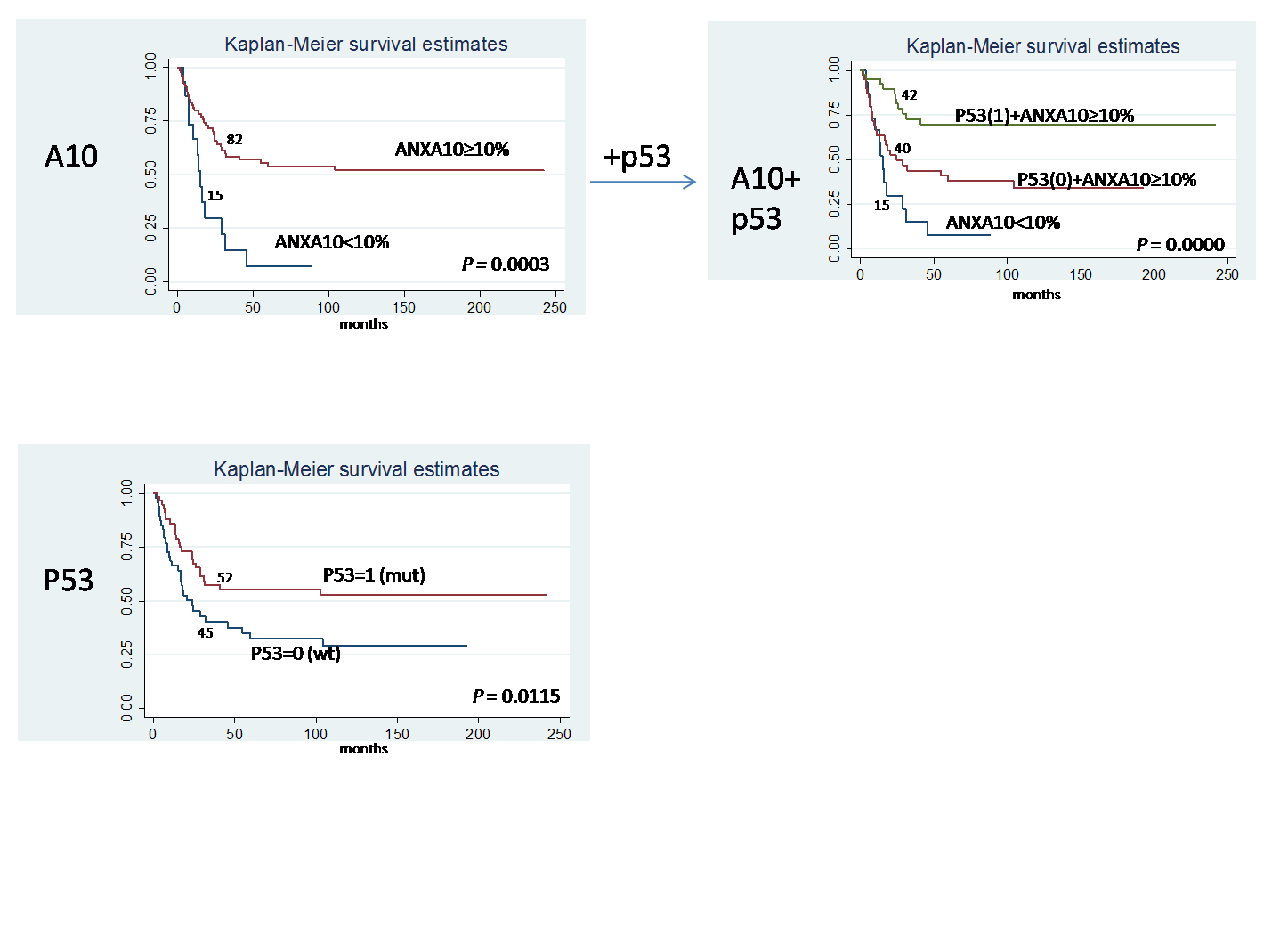


B


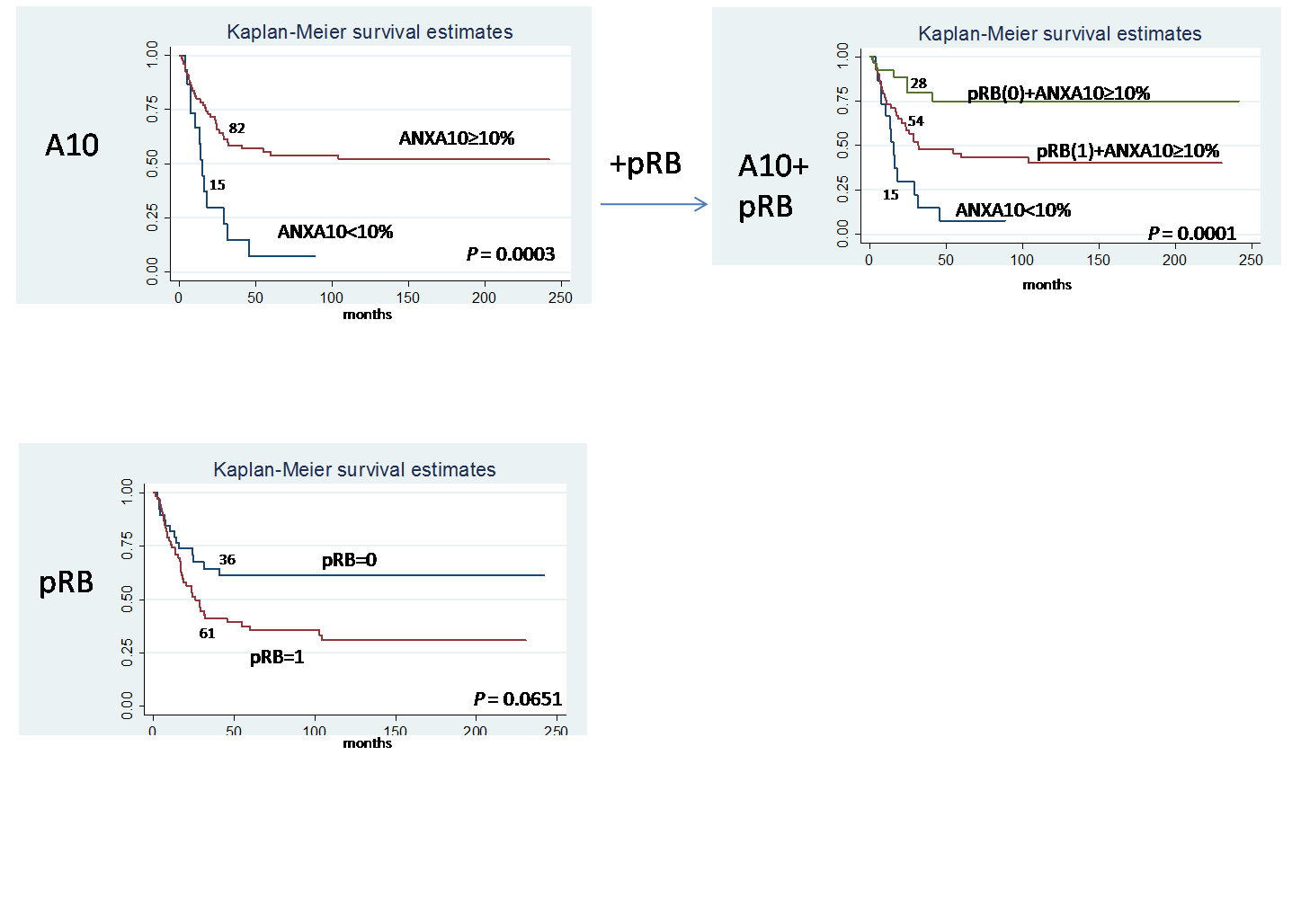

Supplement: Supplementary Information [file bjc2011404x1.doc]
